# Supplementary material for: Overlapping qubits from non-isometric maps and de Sitter tensor networks
Source: Nat Commun. 2025 Jan 2;16:163. doi: 10.1038/s41467-024-55463-9 (PMC11696274; doi:10.1038/s41467-024-55463-9)
Supplement: Supplementary file 1 — Supplementary Information [file 41467_2024_55463_MOESM1_ESM.pdf]

Overlapping qubits from non-isometric maps and de Sitter tensor networks: Supplementary Information

ChunJun Cao,[1,2,3] Wissam Chemissany,[4] Alexander Jahn,[2,5] and Zoltán Zimborás[6,7,8]

[1] *Joint Center for Quantum Information and Computer Science, University of Maryland, College Park, MD, 20742, USA*
[2] *Institute for Quantum Information and Matter, California Institute of Technology, 1200 E California Blvd, Pasadena, CA 91125, USA*
[3] *Department of Physics, Virginia Tech, Blacksburg, VA, 24061, USA*
[4] *David Rittenhouse Laboratory, University of Pennsylvania, Philadelphia, PA 19104, USA*
[5] *Department of Physics, Freie Universität Berlin, 14195 Berlin, Germany*
[6] *QTF Centre of Excellence, Department of Physics, University of Helsinki, P.O. Box 43, FI-00014 Helsinki, Finland*
[7] *Algorithmiq Ltd, Kanavakatu 3C 00160 Helsinki, Finland*
[8] *HUN-REN Wigner Research Centre for Physics, 1525 P.O. Box 49, Hungary*

SUPPLEMENTARY NOTE 1: REMARK 1 AND RELATED DISCUSSIONS

Let us start with

$$\prod_i^{m\le M} Q_p^{(i)}|\psi_p\rangle = VQ^{(m)}V^\dagger \dots VQ^{(1)}V^\dagger V|\psi\rangle.$$

Since $PS|\psi\rangle \approx S|\psi\rangle$, $\forall S$, the above expression trivially simplifies by applying this condition repeatedly and we have $\prod_i^{m\le M} Q_p^{(i)}|\psi_p\rangle \approx V\prod_i^{m\le M} Q^{(i)}|\psi\rangle = V\mathcal{Q}|\psi\rangle$.
Since $\langle\psi_p|\prod_i^{m\le M} Q_p^{(i)}|\psi_p\rangle = \langle\psi|P\mathcal{Q}|\psi\rangle$ and that $\langle\psi|P \approx \langle\psi|$, it follows that

$$\langle\psi_p|\prod_i^{\le M} Q_p^{(i)}|\psi_p\rangle \approx \langle\psi|\prod_i^{\le M} Q^{(i)}|\psi\rangle.$$

These conditions are sufficient for identifying well-spoofed processes, but are not necessary.
For example, in the construction of Akers and Penington [1], the inner product between vectors $|\psi\rangle, |\phi\rangle$ with support mostly in $ker(P)$ is considered (instead of vectors satisfying $P|\psi\rangle \approx |\psi\rangle$ as in our construction).
Then we see in their construction, that thanks to the random nature of V, with high probability

$$\langle\phi_p|\psi_p\rangle = \sum_i \lambda_i \langle\phi|i\rangle\langle i|\psi\rangle \approx \langle\phi|\psi\rangle, \tag{1}$$

where $|i\rangle$ are the eigenstates of P with eigenvalue λ_i.
Here we do not provide a rigorous account for the necessary condition but seek to build up some intuition. For any set of operators $\{Q^{(i)}, i = 1, \dots M\}$,

$$\langle\psi_p|Q_p^{(M)} \dots Q_p^{(1)}|\psi_p\rangle = \langle\psi|PQ^{(M)}P \dots PQ^{(1)}P|\psi\rangle$$
$$= \sum_{i_j; j=0,\dots M} \prod_{j=0}^{M} \lambda_{i_j} \langle\psi|i_M\rangle\langle i_M|Q^{(M)}|i_{M-1}\rangle \dots$$
$$\times \langle i_1|Q^{(1)}|i_0\rangle\langle i_0|\psi\rangle$$

One can think of this expression as a sum of paths with complex weights from $j = 0$ to $j = M$ where at each "time" j the trajectory can take on different values by inserting P instead of I in a conventional path integral. As a result, some path will terminate when they reach the kernel while others stay in the orthogonal complement. For instance, if we take identical operators $Q \sim \exp(i\Delta t H)$ and $\lambda_i \in \{0, 1\}$, this is nothing but the usual propagator but without summing over paths that enter the kernel.
For fixed V and $|\psi\rangle$, the search of $Q^{(i)}$s amounts to solving an approximate constraint satisfaction problem where each $\langle\psi_p|Q_p^{(M)} \dots Q_p^{(1)}|\psi_p\rangle \approx \langle\psi|Q^{(M)} \dots Q^{(1)}|\psi\rangle$ provides a constraint on the matrix elements of $Q^{(i)}$. For a simple example, consider a one-point function $\langle\psi_p|Q_p|\psi_p\rangle \approx \langle\psi|Q|\psi\rangle$ for fixed $V, |\psi\rangle$ and some operator Q with $\lambda_i = 1$ for $i = 1, \dots, \Lambda$ and 0 otherwise.

$$\sum_{i,k} \langle\psi|i\rangle\langle i|Q|k\rangle\langle k|\psi\rangle = \sum_{i,k=1}^{\Lambda} \psi_i Q_{ik} \psi_k \approx \langle\psi|Q|\psi\rangle. \tag{2}$$

This is an approximate constraint on the matrix elements of possible operators Q for which the expectation value is well-spoofed.

SUPPLEMENTARY NOTE 2: NUMERICS ON SPECTRAL PROPERTIES

Here we consider spectral properties of T_{ij} and related operators used for commutators. Recall that $T_{ij} = (V^\dagger V) - I$. For Vs that are proportional to Haar random projections like [1], the individual matrix elements are Gaussian distributed (Figure 1) which we confirm using small size numerics. Note that however, different matrix elements are correlated and the ensemble generated by different Vs is not a Gaussian unitary ensemble. This can be seen by noting that spec(T) has a eigenvalues $2^{N-n}-1$ and -1, as required by the singular value decomposition of V, i.e., spec(P) = spec($V^\dagger V$) has eigenvalues 2^{N-n} for 2^n eigenvectors and 0 for the rest.

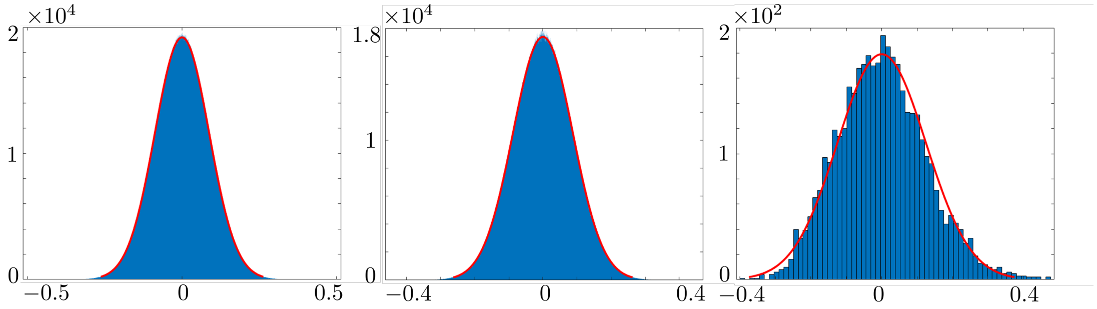

Supplementary Fig. 1. *Off-diagonal projector elements.* Plotting the distribution of off-diagonal elements in T_{ij} for $N = 12, n = 6$. The raw (unfitted) variances of the distributions are $\sigma^2_{Re;off} \approx 0.077, \sigma^2_{Im;off} \approx 0.077, \sigma^2_{diag} \approx 0.0156$. The raw means are $-1.6892e-05, 3.2195e-20, 9.8229e-17$ respectively for the off diagonal real, imaginary parts and the diagonal. Red curves are Gaussian fits. The fitted parameters are $(-1.68924e-05, 0.0877034), (-3.21951e-20, 0.0876864), (9.82287e-17, 0.124988)$ respectively for these 3 data sets.

The behaviour of the commutator, however, is somewhat different. Let us first examine the behaviour of two point functions. Recall that the non-trivial contribution to the projected commutation relation is proportional to $VQTQ'V^\dagger$ where we assume Q, Q' to be local operators acting on different qubits in $\mathcal{H}_N$. By choosing $Q = X_1, Q' = Z_2$ in our numerics, we note the distributions for QTQ' appear similar, except the off-diagonal imaginary parts have a delta function-like peak near 0. Also the diagonal elements now are complex. The delta-function-like peak is likely related to the local Paulis shifting the diagonal of T, which is real. This introduces 2^n identical 0s in the distribution of imaginary components whereas they are non-zero for the real components. Hence the peak only appears in one of the distributions (Figure 2).

For the commutator, the non-trivial contribution comes from $C = QTQ' - Q'TQ$ except its diagonal is purely imaginary now. This is also understandable because we have chosen Q, Q' to be Hermitian. Therefore, iC is Hermitian. The probability distributions for its diagonal and off-diagonal elements are similar to those of the 2 point function, i.e. , they are mostly Gaussian except for the off-diagonal imaginary elements which have a sharp delta function peak near 0. The eigenvalue distributions for $VCV^\dagger$ is more interesting. It is not clear if it is still supposed to follow the semi-circle law. Nevertheless, we do observe a distribution that is mostly concentrated around 0 (Figure 3).

Also for comparison, we can consider a map V that is not Haar random, but is given by the rescaled projection onto the low energy subspace of a 1d critical Ising model

$$H = -\cos\theta \sum_i Z_i Z_{i+1} - \sin\theta \sum_i X_i,$$

where $V = \Pi_{E<\Lambda}\sqrt{2^{N-n}}$ where Λ is the $2^n + 1$th energy eigenvalue and the Hamiltonian has periodic boundary condition.

It is clear then that the distribution of matrix elements is no longer Gaussian (Figure 4). Only real components of C are plotted as imaginary components are 0. Nevertheless, the majority of the values are concentrated around 0 for C and most of the eigenvalues are also vanishing, hence indicating that the local physics is approximately preserved for most states in the (physical) Hilbert space.

Interestingly, if we examine the eigenstates of $|VCV^\dagger|$, the states with smaller eigenvalues (i.e. better approximations of the vanishing commutation relations) also tend to have lower energies compared to the ones that have larger violations. The energy here is computed with respect to the projected Ising Hamiltonian $VH_{\text{Ising}}V^\dagger$. As the projected Hamiltonian need not generate the dynamics on the fundamental Hilbert space, we will refer to this as pseudo-energy instead. More precisely, let $|\phi_k\rangle = |\lambda_p^{|i-j|,k}\rangle$ be the k-th eigenvector of the commutator $[X_p^{(i)}, Z_p^{(j)}]$ with eigenvalue $\lambda_{|i-j|}(k)$ where we have arranged the eigenvalues in ascending order. Then define

$$\omega_{|i-j|}(k) = \langle\lambda_p^{|i-j|,k}|VH_{\text{Ising}}V^\dagger|\lambda_p^{|i-j|,k}\rangle$$

as the pseudo-energy. Then the relation between the eigenvalue $\lambda_\Delta(k)$ and $\omega_\Delta(k)$ is shown in Fig. 3b (main text) where we have also averaged overall positions i with fixed $\Delta = |i-j|$. For this plot, $N = 12, n = 6$ and $\Delta = 5$. Most states preserve the vanishing commutation, but large violation occurs above an energy threshold.

This is intuitive in that we expect lower energy processes to preserve the locality better, while higher energy ones can "back-react" more and hence incurring a bigger correction to the commutation relation. Also note the states with a wide range of different energies preserve the commutation relation.

As a reference, the state-dependent commutator norm $\|[X_p^{(i)}, Z_p^{(j)}]|\psi_p\rangle\|$ also has distance dependence (Fig. 5), like the full commutator norm. Unsurprisingly, it has a smaller overlap compared to the full norm (Fig. 3 in the main text) as the value is taken with respect to a particular state.

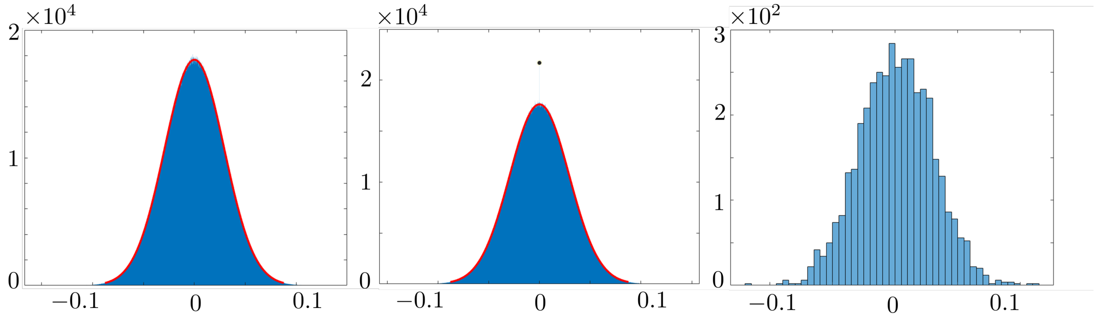

Supplementary Fig. 2. *Real components.* From left to right: distribution of X_1TZ_2' off diagonal real components, off-diagonal imaginary components, and diagonal real components. Imaginary diagonal components have similar distribution. Note the peak in the middle figure near 0 which is barely visible due to its bin width but has height 21696.

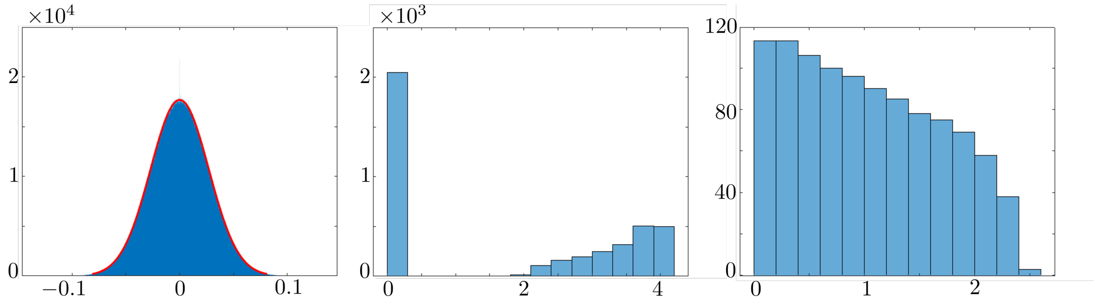

Supplementary Fig. 3. *Imaginary components.* From left to right: imaginary off-diagonal components (note the spike at 0), $|eigenvalue|$ distribution of C, $|eigenvalue|$ distribution of $VCV^\dagger$. The other components of C are have similar distribution to the 2 point function. The real eigenvalue distributions of iC and $iVCV^\dagger$ are symmetric with respect to the y axis, hence the magnitude plot is sufficient.

SUPPLEMENTARY NOTE 3: COMMUTATOR IN GLOBAL DS MERA

First recall that for two observables $O(i), O(j)$ separated by $|i-j|$ sites on the most UV, T-th, "future infinity" layer, their "coarse-grained" IR versions are given by the superoperators induced by their past causal cone. It is clear that $[O(i)_p, O(j)_p]|\psi_p\rangle = 0$ if their past causal cones on V do not intersect.

Other than isolated choice of i, j where the past causal cones never intersect because of the lack of translational symmetry in binary MERA, it generally takes $\sim \log_2|i-j|$ layers before their past causal cones intersect. After these causal cones merge, the subsequent coarse-graining will act on these operators as ascending superoperator with an operator spectrum $\{\lambda_k \leq 1\}$ where $\lambda_0 = 1$ is generically unique for random unitaries. The repeated applications of these superoperators $\mathcal{E}$ slowly shave off the effective target Hilbert space dimension for the remaining $T - \log_2|i-j|$ layers of coarse graining, as the support over smaller eigenvalues will be exponentially suppressed with the number of times the superoperator is applied.

This action of $\mathcal{E}$ acts like a truncation map, but with a soft cut-off. L applications of $\mathcal{E}$ does the following in the eigenbasis

$$\mathcal{E}^L \to \begin{pmatrix} \lambda_0^L & 0 & 0 & \dots \\ 0 & \lambda_1^L & 0 & \dots \\ 0 & 0 & \lambda_2^L & \dots \\ \vdots & \vdots & \ddots & \ddots \end{pmatrix} \tag{3}$$

with $\lambda_1 \geq \lambda_2 \geq \dots$. Instead of introducing a hard cut off above some λ_k, this operator suppresses part of the spectrum with each iteration. Because of this exponential suppression, let us approximate $\mathcal{E}^L : N \to M_L$ as a non-isometric map where M_L, N are the corresponding operator Hilbert spaces and $\dim N \approx \exp(\eta L) \dim M_L$. If we take a generic representative of one such map, it is a random projection that preserves approximate orthogonality. From concentration of measure [2], we know that there exists an embedding of $S_{dS} \sim \log N \sim \exp(\epsilon_L^2 \log M_L)$ qubits into $\log M_L$ exact qubits with $O(\epsilon_L)$ overlap. For random projection, this only occurs with high probability. At $L = 0$, $\log N = 2 \log M_0$ in a binary MERA. Since $L = T - \log|i-j|$, there exists a Johnson-Lindenstrauss-type mapping such that

$$\|[O_p^{(i)}, O_p^{(j)}]\|^2 < \epsilon^2 \tag{4}$$

$$\text{where } \epsilon^2 \sim \frac{\log S}{(-\eta L + S)} \approx \frac{\log S}{\eta \log|i-j| + S - \eta T}. \tag{5}$$

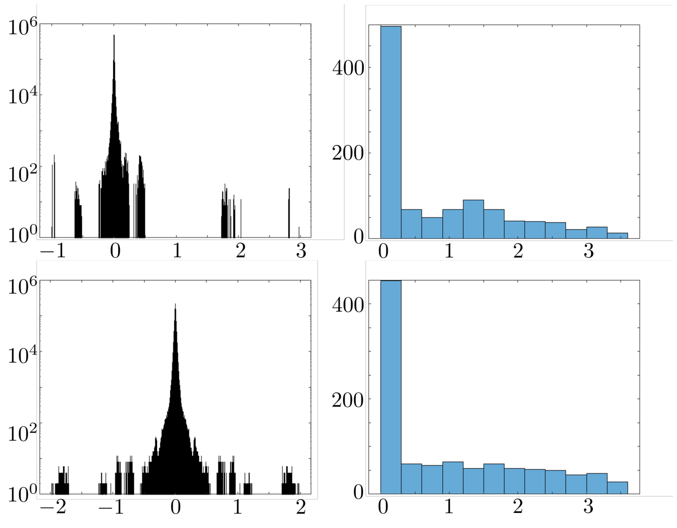

Supplementary Fig. 4. *Non-Gaussian matrix elements.* $N = 12, n = 10$. Left to right: distribution of the value of C when V is given by the rescaled projection onto the low energy subspace; eigenvalue distribution of $|VCV^\dagger|$. Top line: non-critical Ising at $\theta = \pi/5$. Bottom line: critical Ising at $\theta = \pi/4$.

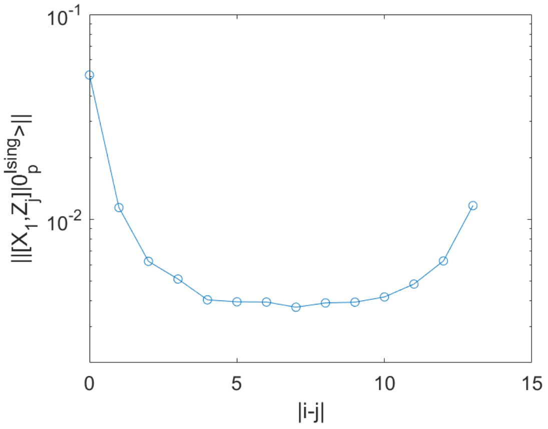

Supplementary Fig. 5. *State-dependent commutator norm.* Value of $\|[X_p^{(i)}, Z_p^{(j)}]|0_p^{Ising}\rangle\|/\ \|X_p^{(i)}\|\|Z_p^{(j)}\|$ plotted with respect to $|i-j|$ for $i = 1$. $|0_p^{Ising}\rangle$ is the projected ground state of a 1d Ising model over $N = 14$ qubits with periodic boundary condition to a smaller $n = 6$ qubit Hilbert space using global energy truncation. The on-site non-commutation is approximately 12 times the smallest overlap.

The largest overlap occurs when $L \sim T$, where the two operators started off in the same static patch. Therefore $\epsilon \sim O(1)$ when $T \sim (S - \log S)/\eta$. Note that this is an estimate for the upper bound for the commutator norm, and therefore a lower bound for the cut off of time T after which locality breaks down.
This time estimate is unsurprising, because there exist known mappings [2] where N constant pair-wise overlapping qubits can be achieved with $n = O(\frac{1}{\epsilon^2}\log(N))$ non-overlapping ones. While it would limit the time of

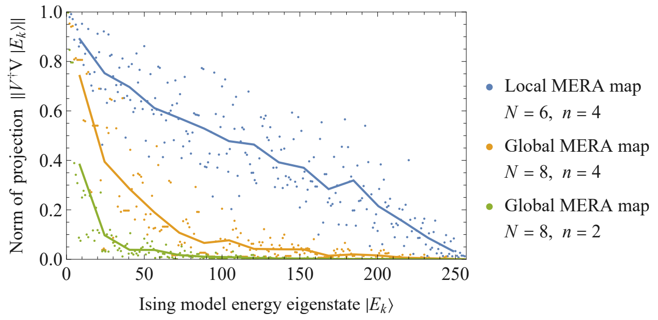

Supplementary Fig. 6. *Energy eigenstate truncation.* Norm of energy eigenstates of the 8-spin critical Ising model, after projection onto the fundamental Hilbert space of the $N = 6, n = 4$ local model (times $\mathbb{1}^{\otimes 2}$ acting on two sites) as well as the $N = 8, n = 4$ and $n = 2$ global model. Eigenstates are ordered by their energy eigenvalues. The solid curve shows the average fidelity of blocks of 16 eigenstates.

exponential expansion to $t \sim n \sim S_{dS}$, from a quantum cosmology perspective, the above requirement is still too strong because the Universe is described by a single state vector. Therefore we are more interested in the overlap with respect to a particular state, which we look at now. Note that the channel $\mathcal{E}^L$ is produced by a state level non-isometric map supplied by the MERA tensor network such that $W : \mathcal{H}_{L_{int}} \otimes \mathcal{H}_{L_{hor}} \to \mathcal{H}_{0_A}$ maps from the interior and horizon degrees of freedom of the causal patch at $t = L$ to a subregion A of the Σ_0 slice after the past causal cones of $O^{(i)}, O^{(j)}$ merge. This map does not treat all inputs equally as they are introduced at different layers. Like before, let us suppose that the action of this map on the $\mathcal{H}_{L_{int}}$ subspace can be approximated by a random projection $W' : \mathcal{H}_{L_{int}} \to \mathcal{H}_{M_L} \subset \mathcal{H}_{0_A}$ such that $d_{M_L} = |\mathcal{H}_{M_L}| = 2^{-\eta' L}|\mathcal{H}_{L_{int}}| \sim 2^{-\eta' L}e^S$. Let $d_{L_{int}} = |\mathcal{H}_{L_{int}}|$. Using a random projection, this allows us to embed $\mathcal{H}_{L_{int}}$ into $\mathcal{H}_{M_L}$ with $d_{L_{int}} \sim \exp(\epsilon_L'^2 d_{M_L})$. It is easy to show that $\|[O(i)_p, O(j)_p]|\psi_p\rangle\| \leq \epsilon'\|T_p^{[O(i),O(j)]}|\psi_p\rangle\|$ where the matrix elements of $T^{[O(i),O(j)]}$ is of at most $O(1)$ with high probability and

$$\epsilon'^2 \approx \frac{\log(S)e^{-S}e^{\eta' T}}{|i-j|^{\eta'}}.$$

Thus ϵ' becomes $O(1)$ for $T \sim (S - \log\log S)/\eta'$.
Therefore, we expect the overall estimate for locality to break down in a time scale similar to the state-independent case. Furthermore, one should expect $\|[Q_i, Q_j]|\psi_p\rangle\| \lesssim O(\tilde{\epsilon}/|i-j|^{\eta'/2})$ for fixed S, T to satisfy a power law as long as the approximation for the map W is correct (Fig 5).
This is not sufficient to show that the norm is small, however, as T_p is generally not unitary. We would also need there to exist $|\psi_p\rangle$ such that $\|T_p^{[O(i),O(j)]}|\psi_p\rangle\|$ does not grow exponentially with S. Although numerically we can verify that $\mathrm{spec}(T_p^{[O(i),O(j)]})$ has a large number of low lying eigenvalues (Appendix) where the minimum eigen-

value appears stable at different S's, it is insufficient for any asymptotics. There is also no reason to expect that random tensors are physically relevant other than technical convenience. We leave a full analysis of $T_p^{[O(i),O(j)]}$'s spectral properties for local operators O to future work. Instead of W', we can construct a slightly different map $V : \mathcal{H}_{L_{int}} \to \mathcal{H}_{M_L}$ with a bit more structure by choosing a different set of disentanglers and isometries.
Let us define

$$\begin{aligned}
&\mathcal{D}_0 = \{|\psi\rangle\} \subset \mathcal{H}_{L_{int}},\\
&\mathcal{D}_1 = \mathrm{span}\{Q_i|\psi\rangle, \forall\, Q_i\} \cap \mathcal{D}_0^{\perp}, \ldots\\
&\mathcal{D}_M = (\bigoplus_{i=0}^{M-1} \mathcal{D}_i)^{\perp}\\
&\qquad \cap \mathrm{span}\{Q_{i_1} \ldots Q_{i_M}|\psi\rangle, \forall\, Q_{i_j} : i_j \neq i_k \text{ if } j \neq k\}.
\end{aligned}$$

It is helpful to think of $\mathcal{D}_i$ as the space of states where the computational basis states have Hamming weight i. Consider a non-isometric map $V = \bigoplus_i V_i$ where each V_i is a random projection from $\mathcal{D}_i$ to $\mathcal{D}_i' \subset \mathcal{H}_n$ such that $V^\dagger V$ preserves the vector inner product approximately within each block but exactly across different blocks. Define $\mathcal{C}_r = \bigoplus_{i=0}^r \mathcal{H}_i$ and $C_r = |\mathcal{C}_r|$. Using this map, we embed $\mathcal{C}_M$ into a $O(e^{S-\eta' T})$ dimensional subspace of the physical Hilbert space $\mathcal{H}_{0_A}$ such that orthogonality within each subspace $\mathcal{H}_i$ is approximately preserved using the JL theorem up to overlap bounded by ϵ, and have the remaining states mapped to the null space. By the direct sum, orthogonality across different subspaces are exactly preserved.
Then

$$\begin{aligned}
&Q_p^{i_M} \ldots Q_p^{i_2} Q_p^{i_1}|\psi_p\rangle = Q_p^{i_M} \ldots V Q^{i_2} V^\dagger V Q^{i_1}|\psi\rangle\\
&= Q_p^{i_M} \ldots V Q^{i_2} Q^{i_1}|\psi\rangle + \epsilon Q_p^{i_M} \ldots V Q^{i_2} T Q^{i_1}|\psi\rangle\\
&= V(Q^{i_M} \ldots Q^{i_1}|\psi\rangle + \sum_{k=1}^{M-1} \epsilon^k O_k[Q,T]|\psi\rangle)
\end{aligned}$$

where $O_k[Q,T]$ is the set of all operator strings consisting of $Q^{i_M} \ldots Q^{i_1}$ where total k T operators are inserted into the $M-1$ gaps between Q operators. Only a single T may be inserted for each gap. Note that

$$\|O_k[Q,T]|\psi\rangle\| \leq C_{M-1}^k$$

because T by construction is block diagonal, hence T applied to any state in $\mathcal{C}_r$ never maps it to a state outside this subspace, unlike the fully Haar random construction. Since $|\mathcal{D}_i| = \binom{S}{i} \sim S^i$, $C_{M-1} \leq M S^{M-1}$ which is polynomial for bounded M. Then

$$\epsilon C_{M-1} \lesssim \frac{S^{M-1} M^{3/2} \sqrt{\log S}}{e^{(S-\eta' L)/2}} \ll 1,$$

for fixed M, $S \gg 1$ and $L \lesssim (S - O(M\log S) - \log M)/\eta'$. Recall that $V^\dagger V|\psi\rangle = |\psi\rangle$, therefore any M-point function can be approximated with the above restrictions

$$\begin{aligned}
&|\langle\psi_p|Q_p^{i_M} \ldots Q_p^{i_1}|\psi_p\rangle - \langle\psi|Q^{i_M} \ldots Q^{i_1}|\psi\rangle|\\
&\leq \sum_{k=1}^{M-1} |\epsilon^k \langle\psi|V^\dagger V O_k(Q,T)|\psi\rangle| \leq \sum_{k=1}^{M-1} \left(\epsilon \binom{M-1}{k} C_{M-1}\right)^k\\
&< \sum_{k=1}^{M-1} (\epsilon M C_{M-1})^k \ll 1.
\end{aligned}$$

Thus for operators within the same static patch, this approximation is valid up to $T = L \lesssim O(S - M\log S - \log M)$. Because the state-dependent commutator norm $\|[Q_p^{i_1}, Q_p^{i_2}]|\psi_p\rangle\|$ can be expanded as a sum of 4 point functions, its values are also preserved for the same amount of time before the correction becomes order 1.
For commutators of operators Q^{i_1}, Q^{i_2} that live in different static patches, we have $T = L + \log|i-j|$, and the correction terms are again $O(\epsilon M C_1) \approx \tilde{\epsilon}/|i-j|^{\eta'/2}$ where $\tilde{\epsilon} \approx S \log S \exp(-S/2 + \eta' T/2)$. Again, this value becomes order one in time at most linear in S.
Assumptions used to produce the above estimates for MERA may fail. For reference, let us also examine a simpler case where we allow constant pairwise overlap using the same type of random map $V : \mathcal{H}_{N_t} \to \mathcal{H}_n$ globally. This simplification removes the local structure of the MERA and is more similar to the one by [1]. We use it as an estimate to bound the longest possible time that a spoofing of local physics can last if N increases exponentially in time. At time T, the apparent Hilbert space consists of $\sim e^T S$ qubits. If we repeat the same exercise and embed the C_M subspace into the physical Hilbert space of dimension e^S using the direct sum of JL mappings, then one can show that

$$\epsilon \sim e^{-S/2}\sqrt{T + \log S},\ C_M \leq (M+1) e^{MT} S^M.$$

For any $M > 1$, the size of the correction scales polynomially with ϵC_{M-1}, meaning that it is small for at most $T \lesssim S/2M$. This is consistent with our previous estimate for the amount of time needed before such local physics breaks down. However, for $M = 1$, $C_0 = 1$ and the exponential multiplicative factor does not enter. Hence a two point function with respect to the special "vacuum" state $|\psi\rangle$ has

$$\begin{aligned}
&\langle\psi_p|Q_p^{i_1} Q_p^{i_2}|\psi_p\rangle = \langle\psi|Q^{i_1} V^\dagger V Q^{i_2}|\psi\rangle\\
&= \langle\psi|Q^{i_1} Q^{i_2}|\psi\rangle + O(\epsilon).
\end{aligned}$$

Hence the correction here only becomes order 1 when $T \sim e^S - \log S$, which roughly coincides with the time it takes for the proposed dS dynamics to reach maximum complexity [3].

SUPPLEMENTARY NOTE 4: CRITICAL ISING AND HAAR RANDOM MERA

In the main text, we consider a MERA tensor network of bond dimension $\chi = 2$ with tensors chosen to approximate the critical Ising model. Here we present the details

of this construction: As shown in Fig. 5 (main text), the two types of MERA tensors are 3-leg isometries and 4-leg unitaries (or *disentanglers*). We can represent these tensors by a 2×4 matrix M_i and a 4×4 matrix M_d, respectively. The Ising model at its critical point is given by Hamiltonian

$$H_{\rm I} = -\sum_{k=1}^{N} \left(X_k X_{k+1} + Z_k \right) \ , \tag{6}$$

where X_k, Z_k are Pauli operators acting on the kth site in a chain of N spins. Its ground state can be solved using free-fermion techniques, and so it is convenient to use MERA tensors that are themselves free fermion operators. An ansatz for M_i and M_d that fulfills the isometry/unitary constraint $M_i^\dagger M_i = M_d^\dagger M_d = \mathbb{1}$ and is free-fermionic is given by [4]

$$M_i = \begin{pmatrix} \cos\alpha & 0 & 0 & \sin\alpha \\ 0 & \frac{1}{\sqrt{2}} & -\frac{1}{\sqrt{2}} & 0 \end{pmatrix} \ , \tag{7}$$

$$M_d = \begin{pmatrix} \cos\beta & 0 & 0 & \sin\beta \\ 0 & 1 & 0 & 0 \\ 0 & 0 & -1 & 0 \\ -\sin\beta & 0 & 0 & \cos\beta \end{pmatrix} \ , \tag{8}$$

where α, β are real angles. Note that our M_d differs slightly from the one proposed in [4], though both are valid solutions. We also need to specify an initial (central) 4-leg tensor T^0. A T^0 that represents a free fermion state with minimal energy with respect to (6) can be found analytically [5] and has nonzero entries

$$T_{1,1,1,1} = \frac{1}{N} \ , \tag{9}$$

$$T_{2,2,1,1} = T_{1,2,2,1} = T_{1,1,2,2} = T_{2,1,1,2} = \frac{a_0}{N} \ , \tag{10}$$

$$T_{2,1,2,1} = T_{1,2,1,2} = \frac{b_0}{N} \ , \tag{11}$$

$$T_{2,2,2,2} = \frac{2a_0^2 - b_0^2}{N} \tag{12}$$

where

$$N = \sqrt{1 + 4a_0^2 + 2b_0^2 + (2a_0^2 - b_0^2)^2} \ , \tag{13}$$

with $a_0 \approx 0.3066$, $b_0 \approx 0.2346$. Given this initial tensor, one can then find the α, β that best approximate the critical Ising ground state. For a MERA with two layers describing $N = 16$ sites, we perform a numerical minimization using a matchgate tensor network [5] to find $\alpha \approx 1.831, \beta \approx 1.682$. This corresponds to a ground state energy of ≈ -0.6358 per site, very close to the exact value $-\frac{2}{\pi} \approx -0.6366$ for the critical Ising model in the continuum limit.
For both the global and local MERA maps $V : \mathcal{H}_N \to \mathcal{H}_n$ considered in the main text, we can build a projector $P = V^\dagger V$ onto the "fundamental subspace" of $\mathcal{H}_N$ using the explicit tensor network form of V. We then compute the

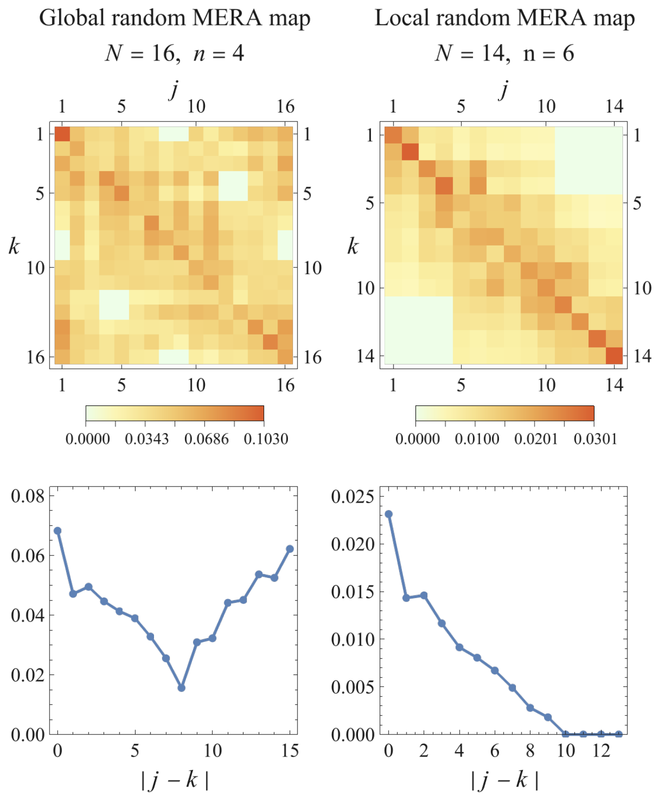

Supplementary Fig. 7. *Random MERA overlaps.* Commutator trace norms $\|[\tilde{X}_j, \tilde{Z}_k]\|_1 / \|\tilde{X}_j\|_1 \|\tilde{Z}_k\|_1$ of projected Pauli operators as in Fig. 7, but for the global and local MERA map with Haar-random isometries and disentanglers. The plots are averages of five samples, with tensors chosen locally random in each sample. The second row shows the average decay with distance $|j - k|$.

overlap of this subspace with the energy eigenbasis of the Ising Hamiltonian (6). The result, shown in Fig. 6, is that both the global and local MERA preserve the low-energy part of the spectrum. While the global MERA truncation is sharp (as expected from the IR/UV interpretation of the MERA layers), the local MERA truncation gradually loses fidelity as one moves from lower- to higher-energy states.
Rather than the non-interacting Ising model, we consider a second numerical setup in which the MERA tensors are locally chosen Haar-randomly. Specifically, that means that each disentangler is chosen as an independently Haar-random unitary map acting on two qubits, while each isometry is chosen by the projection of such a Haar-random unitary on the $|0\rangle$ state of one of the two output qubits, resulting in an isometric map. The norm of the resulting commutators is shown in Fig. 7, and follows similar qualitative features as the Ising case, suggesting that the MERA geometry and tensor restrictions, by themselves, ensure an approximate locality in the truncated Hilbert space. Note that while we only consider local (bond) dimension $\chi = 2$, such a construction can easily be generalized to any larger χ, serving as

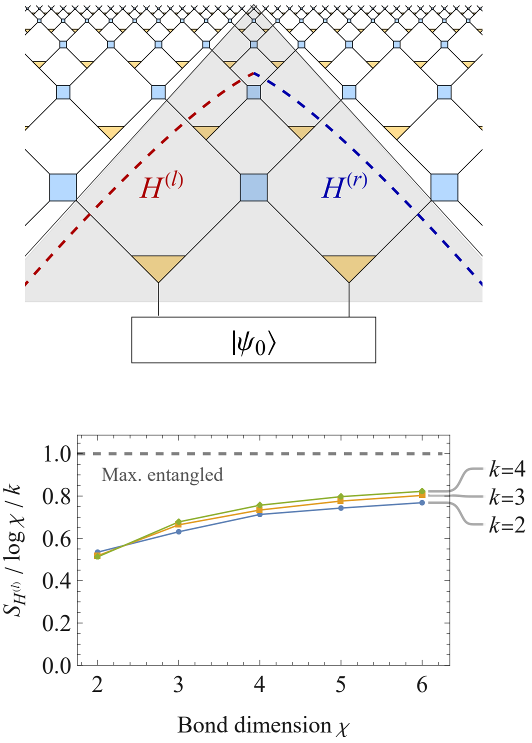

Supplementary Fig. 8. *Entanglement along the static patch horizon.* We compute entanglement for the MERA discretization of dS_2. We consider an initial state $|\psi_0\rangle = |0\rangle^{\otimes 2}$ and apply two isometries and one disentangler in each layer. We approximate the static patch horizon by terminating after k layers, here shown for $k = 2$, and compute the entanglement entropy $S_{H^{(l)}}$ between the left and right half $H^{(l)}$ and $H^{(r)}$. Shown below is the result for Haar-random unitaries and isometries of bond dimension χ, with apparent convergence to maximal entanglement as $\chi, k \to \infty$.

a toy model for higher-dimensional CFT states.

SUPPLEMENTARY NOTE 5: ENTANGLEMENT IN THE STATIC PATCH

As discussed in the main text, the local MERA map (Fig.2(b) in the main text), when applied to the exterior region between the static patches of two antipodal observers in dS_2, relates the Hilbert space on (half of) the static patch horizons to that of a time-slice of the exterior region. This raises the following question: If we choose the MERA tensors to well approximate the ground state of a critical theory, and hence produce a subsystem reduction ρ_R of such a state on a time-slice of the exterior region, what is the dual state $\tilde{\rho}_{H_R} \equiv \tilde{\rho}_R$ on the horizon degrees of freedom produced by applying the local MERA map? This density matrix $\tilde{\rho}_R$ can be constructed by contracting all of the tensors *outside* of the local wedge $\mathcal{W}_R$. By symmetry, this corresponds to contracting the tensors within each static patch. For generic tensors, such as those describing the bond dimension $\chi = 2$ MERA approximation of the critical Ising model, $\tilde{\rho}_R$ will describe a state with complicated entanglement. The entanglement along the horizon can, however, be bounded by $|H_R| \log \chi$, $|H_R|$ being the number of sites along the horizon H_R, which we make finite by a time-slice cutoff at finite t. This argument is exactly analogous to the Ryu-Takayanagi entanglement entropy bound in previous AdS/MERA proposals [6], where H_r takes the form of the RT surface γ_R [7]. As in AdS/MERA, we expect our tensor network to represent gravitational features only in the limit of very large bond dimension χ. Choosing such a limit for random tensors, one finds that the entanglement entropy saturates the upper bound $|\gamma_R| \log \chi$, i.e., exhibits maximal entanglement across minimal cuts γ_R [8]. Applying this observation to our dS picture, we expect that for a MERA tensor network with Haar-random disentanglers and isometries at large bond dimension, the entanglement along H_R will asymptote to $|H_R| \log \chi$. Within each static patch, this implies that the left and right "half" of each horizon become maximally entangled with each other, as the minimal cut for each half coincides with the sites themselves. We show the slow convergence to this limit numerically in Fig. 8. This leads to a horizon state $\tilde{\rho}_R \propto \mathbb{1}$, i.e., the maximally mixed state. This behavior is striking, as it implies that the local MERA map V relates the entanglement spectrum of a CFT ground state (assuming such can be approximated by high bond dimension MERA) to a completely flat one characteristic of maximal entanglement. V thus appears to distill the complicated entanglement between a CFT subregion R and its complement into EPR pairs. This behavior has interesting consequences in the continuum limit, where the partial trace of QFT subregions is ill-defined due to divergences in the entanglement spectrum formally associated with a type III von Neumann algebra. However, it has recently been proposed that gravity effects in de Sitter spacetime effectively reduce the algebra of observables in the static patch to one of type II_1, where notions of partial traces and entanglement entropies are well-defined [9]. There are some similarities between this approach and our work, in particular our relation between a maximum-entropy state on one side and and a CFT ground state on another, though the latter is located in the exterior region in our model, rather than in the static patch itself. The more precise behavior of the continuum limit of our local and global MERA maps is an interesting area for future work.

[1] C. Akers and G. Penington, arXiv preprint arXiv:2109.14618 (2021).

[2] R. Chao, B. W. Reichardt, C. Sutherland, and T. Vidick, 8th Innovations in Theoretical Computer Science Confer-

ence (ITCS 2017) (Schloss Dagstuhl – Leibniz-Zentrum für Informatik, 2017) pp. 48:1–48:21, arXiv:1701.01062 [quant-ph].
[3] L. Susskind, arXiv preprint arXiv:2106.03964 (2021).
[4] G. Evenbly and S. R. White, Phys. Rev. Lett. **116**, 140403 (2016), arXiv:1602.01166 [cond-mat.str-el].
[5] A. Jahn, M. Gluza, F. Pastawski, and J. Eisert, Sci. Adv. **5**, eaaw0092 (2019), arXiv:1711.03109 [quant-ph].
[6] B. Swingle, Phys. Rev. D **86**, 065007 (2012), arXiv:0905.1317 [cond-mat.str-el].
[7] S. Ryu and T. Takayanagi, Phys. Rev. Lett. **96**, 181602 (2006), arXiv:hep-th/0603001.
[8] P. Hayden, S. Nezami, X.-L. Qi, N. Thomas, M. Walter, and Z. Yang, JHEP **11**, 009 (2016), arXiv:1601.01694 [hep-th].
[9] V. Chandrasekaran, R. Longo, G. Penington, and E. Witten, arXiv preprint arXiv:2206.10780 (2022).
